# Trait-Level Variability in Attention Modulates Mind Wandering and Academic Achievement

**DOI:** 10.3389/fpsyg.2020.00909

**Published:** 2020-05-28

**Authors:** Effie J. Pereira, Lauri Gurguryan, Jelena Ristic

**Affiliations:** Department of Psychology, McGill University, Montreal, QC, Canada

**Keywords:** mind wandering, attention, attentional control, temperament traits, academic achievement

## Abstract

Although mind wandering remains ubiquitous in daily life, the processes that underlie and sustain this behavior remain poorly understood. Across two experiments, we studied the role of intrinsic temperament traits, which shape stable behavioral processes, in moderating the association between mind wandering and the real-life functional outcome of academic success. In Experiment 1, participants completed the Mind Wandering Questionnaire, the Adult Temperament Questionnaire, and reported their grade for the highest degree completed or in progress. Individuals with traits of low Effortful control, high Negative affect, and low Extraversion indicated more mind wandering. Effortful control moderated the relationship between mind wandering and academic success, with higher tendency for mind wandering associated with higher academic achievement for individuals with high Effortful control, and lower academic achievement for those with low Effortful control. Experiment 2 confirmed these links using the visual metronome response task, an objective measure of mind wandering. Together, these results suggest that the intrinsic temperament trait of Effortful control represents one of the key mechanisms behind the functional influence of mind wandering on real-life outcomes. This work places an innate ability to control attention at the very core of real life success, and highlights the need for studying mind wandering through an interdisciplinary lens that brings together cognitive, biological, social, and clinical theories in order to understand the fundamental mechanisms that drive this behavior.

## Introduction

Many of us have experienced periods of inattention when our mind moves away from a primary task onto other thoughts. This behavior, often called *mind wandering*, is ubiquitous in daily life and is estimated to occupy anywhere between 20 to 50% of our waking hours (Killingsworth and Gilbert, 2010; Schooler et al., 2011). A large body of evidence converges on the notion that increased mind wandering has a negative impact on task performance, with detriments observed across a range of tasks, including reading comprehension (Unsworth and McMillan, 2013), verbal reasoning (Mrazek et al., 2012), working memory (Kane et al., 2007), and sustained attention (Cheyne et al., 2009). In addition, performance detriments have also been found during real-life activities, in which the effects of increased mind wandering manifest as deficits in memory retention (Risko et al., 2012), increased fidgeting during lectures (Carriere et al., 2013), and decreased performance on standardized tests (i.e., SATs) (McVay and Kane, 2012; Unsworth et al., 2012). Given the importance of these life consequences, it becomes increasingly important to gain a more in-depth understanding of the mechanisms that underlie and sustain such inattentive behaviors. Recent research has identified personality dimensions as one possible pervasive factor involved in the maintenance of mind wandering (Jackson et al., 2013; Smeekens and Kane, 2016; Kane et al., 2017). Across two experiments, in which we have assessed mind wandering subjectively (Experiment 1) and objectively (Experiment 2), we examined the influence of innate biologically-primed temperament traits on mind wandering and the real-life functional outcome of academic achievement.

Successful performance on many tasks is predicated on prolonged attentional focus on external content and an effective regulation of mind wandering (Szpunar et al., 2013). During effective learning, attention remains focused on external content and is “coupled” with the internal mental processes of working memory, such that information available in the environment is integrated with existing knowledge and mental representations (Smallwood et al., 2007). In contrast, when the mind wanders, attention is directed away from external stimuli and becomes engaged with internal thoughts, memories, and feelings (Schooler et al., 2011). During such a state of “decoupled” attention, attention moves away from the external environment and ceases to aid in the integration of external information with internal representations. Thus, a precursor for successful task performance involves attentional systems effectively integrating information from the external world with the content generated by internal mental processes.

This key role of attentional mechanisms in maintaining effective task performance suggests that an individual’s ability to control attention may be intimately tied to their propensity for mind wandering. Two lines of evidence support this idea. One, mind wandering and attention generally function in a coupled manner but engage opposing ends of the attentional control spectrum. Both behavioral and neuroimaging studies support this notion. Behavioral data show that lower working memory capacity, which is associated with a decreased ability to control attention, is related to increased mind wandering (Kane et al., 2007). Similarly, rates of mind wandering during go/no-go tasks have been related to performance detriments for both go and no-go stimuli (Cheyne et al., 2009; Stawarczyk et al., 2013). Likewise, neuroimaging work has shown that attentional states and mind wandering are associated with dissociable patterns of activity in two large-scale cortical networks, one linked to the control of attention (i.e., the frontoparietal attention network) (Fox et al., 2005; Van Calster et al., 2017) and the other linked to mind wandering states (i.e., the default network) (Raichle et al., 2001; Mason et al., 2007; Smallwood et al., 2008; Christoff et al., 2009; Reichle et al., 2010).

The second line of work that supports the idea that attentional control is strongly related to mind wandering is reflected in the finding that both an ability to control attention and mind wandering behaviors remain relatively stable within individuals and across situations, such as for laboratory versus real-world estimates (McVay et al., 2009; McVay and Kane, 2012; Wammes et al., 2019). This suggests that each individual’s degree of attentional control and their associated prevalence of mind wandering may reflect the workings of pervasive stable influences. Indeed, pioneering work from Singer and colleagues first proposed this idea by associating individual personality factors of openness, conscientiousness, and neuroticism with different mind wandering styles (Singer and McCraven, 1961, 1962; Zhiyan and Singer, 1997). Results from more recent research support these findings, indicating that these intrinsic factors play a role in the stability of attentional styles (Irons and Leber, 2018) and the maintenance of mind wandering behaviors (Jackson et al., 2013; McMillan et al., 2013; Smeekens and Kane, 2016). For example, Kane et al. (2017) found that a combination of personality and contextual factors – such as neuroticism in laboratory tasks and openness in daily life activities – predicted individual differences in the frequency of mind wandering. This work indicates that stable factors within individuals may underlie aspects of attentional control and mind wandering behaviors.

Although previous research has associated personality factors with mind wandering, these factors are often understood as a manifestation of underlying temperament traits (Buss and Plomin, 1984; Gagne, 2001). Temperament reflects innate biologically-primed sensitivities that shape stable behavioral processes, forming the core of personality outcomes as an individual develops within their social, educational, and cultural environment (Rothbart and Derryberry, 1981, 2002). Although there are several major theories of temperament (Goldsmith et al., 1987; Shiner et al., 2012). Rothbart (2007); model is particularly pertinent for investigations of mind wandering and attention because it positions the cognitive ability of attention as a superordinate self-regulatory mechanism which, through interactions between genes and the environment, helps to shape persistent behavioral styles (Ristic and Enns, 2015). As such, this model provides the necessary theoretical context for investigations of how individual differences in innate regulatory capacities impact a person’s propensity to mind wander and related life outcomes. In this model, Rothbart (2007) conceptualizes temperament to vary along the Cognitive-Attentional and Motivational-Emotional axes. The Cognitive-Attentional domain includes the traits of Effortful control, defined as the ability to focus attention and resist distractions, and Orienting sensitivity, defined as reactivity to low intensity information from the self and the environment. The Motivational-Emotional domain includes the traits of Negative affect, defined as sensitivity to negative emotions, and Extraversion, defined as sensitivity to positive experiences and interactions with others. The temperament traits within this model parallel the popular Big Five factor model of personality (Barrick and Mount, 1991), with direct overlap in dimensions as depicted in Table 1 (Evans and Rothbart, 2007; Rothbart, 2007). While past work has found links between mind wandering and personality variables that reflect attentional abilities (Jackson et al., 2013; Forster and Lavie, 2014; Smeekens and Kane, 2016; Kane et al., 2017; Ralph et al., 2017), it remains unknown whether an intrinsic trait-level ability to control attention influences mind wandering and modulates real-life functional outcomes.

**TABLE 1:** Rothbart’s temperament model and the Big Five model of personality.

Rothbart temperament dimension	Big Five personality dimension
Effortful control	Conscientiousness
Orienting sensitivity	Openness; Agreeableness
Negative affect	Neuroticism
Extraversion	Extraversion

Thus in the present study, we assessed mind wandering both subjectively and objectively across two experiments and examined how measures of mind wandering related to innate temperament traits and how this relationship affected the real-life functional outcome of academic achievement. We hypothesized that individual variability in temperament traits, particularly along the Cognitive-Attentional trait of Effortful control, would reliably relate to frequency of mind wandering and significantly modulate academic achievement. This link would provide new evidence for the influence of innate trait-level differences in attentional control in both mind wandering and functional real-world outcomes.

## Experiment 1

### Materials and Methods

#### Participants

**FIGURE 1:**
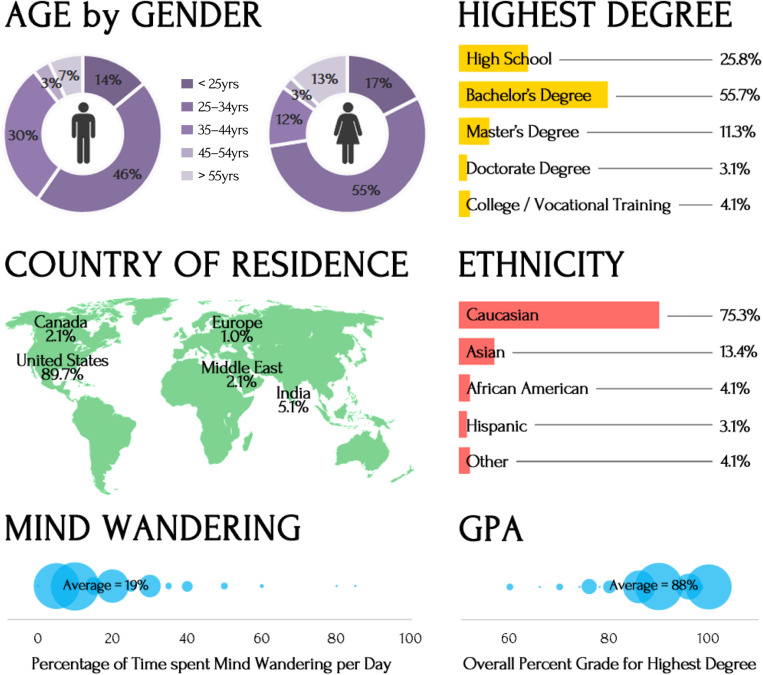
Demographic information.

One hundred and twenty-eight participants were recruited via Amazon Mechanical Turk. The data from ninety-seven participants (57M; 40F; M_age_ = 35yrs, SD_age_ = 11yrs) who completed the study in full were analyzed^1,2^. Their demographic information is depicted in Figure 1. Written informed consent was obtained from each participant in accordance with the Declaration of Helsinki. All procedures were approved by the University Research Ethics Board. Each participant received $2.

#### Stimuli and Design

Participants were asked to report their demographic information, to indicate their highest academic degree completed or in progress, along with their overall grade achieved (in percent), and to self-report the percentage of time they spent mind wandering per day. Then, they proceeded to complete the Mind Wandering Questionnaire (Mrazek et al., 2013) and the short-form Adult Temperament Questionnaire (Evans and Rothbart, 2007). The task took approximately 20 min.

##### Academic performance

Academic achievement was assessed by asking participants “*What was your cumulative achievement grade (%) in the highest degree obtained or degree currently in progress?*” Overall grade average has been used extensively in past research on mind wandering (Lindquist and McLean, 2011; Risko et al., 2012; Unsworth et al., 2012; Wammes et al., 2016), making it a straightforward performance variable to measure the real life consequences of this behavior.

##### Self-reported mind wandering

Self-reported estimates of mind wandering were assessed by directly asking participants “*On any given day, approximately what percentage of your day do you spend thinking about something other than what you are doing?*” This measure was included to ensure that scores on the Mind Wandering Questionnaire were reflective of participants’ own perception of mind wandering. Subjective measures of mind wandering are often used in the literature, with past research showing that individuals can provide reliable reports of episodes of mind wandering (Christoff et al., 2009; Risko et al., 2012; Seli et al., 2014).

##### Mind wandering questionnaire (MWQ)

The MWQ (Mrazek et al., 2013) is a 5-item self-report scale, which has been found to yield a reliable estimate of an individual’s propensity to mind wander during typical activities (Cronbach’s α = 0.85). As such, the MWQ is meant to capture broader aspects of how mind wandering might arise and be maintained, resulting in an intrinsic measure of mind wandering that is independent of task and awareness. The questionnaire uses a 6-point Likert scale, ranging from 1- *Almost Never* to 6- *Almost Always*, with participants rating each item based on how often they experienced the particular situation (e.g., *“I have difficulty maintaining focus on simple or repetitive work”* or “*I find myself listening with one ear, thinking about something else at the same time*”). Higher scores denote higher estimates of mind wandering. This questionnaire has been widely used in studies assessing individuals’ propensity for mind wandering (Kajimura et al., 2016; Luo et al., 2016) and has yielded estimated rates of mind wandering comparable to those obtained in experimental tasks (Mrazek et al., 2013).

##### Adult temperament questionnaire (ATQ)

The short-form ATQ (Rothbart et al., 2003; Evans and Rothbart, 2007) is a standardized 77-item measure (Cronbach’s α = 0.80). It yields a score for the traits of Effortful control (Cronbach’s α = 0.75) and Orienting sensitivity (Cronbach’s α = 0.84) on the Cognitive-Attentional axes and the traits of Negative affect (Cronbach’s α = 0.72) and Extraversion (Cronbach’s α = 0.70) on the Motivational-Emotional axes. Participants rate each response item (e.g., *“I can keep performing a task even when I would rather not do it”* from Effortful control; *“I’m often aware of the sounds of birds in my vicinity”* from Orienting sensitivity; *“Sometimes minor events cause me to feel intense sadness”* from Negative affect; *“I like conversations that include several people”* from Extraversion) on how well it describes them using a 7-point Likert scale ranging from 1- *Extremely Untrue* to 7- *Extremely True*. Higher scores indicate stronger alignment with a specific trait. The ATQ has been validated across diverse samples (Wiltink et al., 2006; Evans and Rothbart, 2007; Laverdière et al., 2010) and has been found to correlate predictably with personality characteristics (Rothbart and Bates, 1998; Caspi, 2000).

### Results

First, we confirmed that there was a positive relationship between self-report estimates of mind wandering and mind wandering scores on the MWQ to ensure overlap between these two measures. The data supported this notion, with a positive correlation between self-reported and measured mind wandering, *Pearson r*(95) = 0.31, *p* = 0.002. Self-reported percentage of time spent mind wandering per day was estimated at 19% (*SD* = 16%, range = 0–85%), which dovetails with previously reported mind wandering ranges (Killingsworth and Gilbert, 2010; Schooler et al., 2011).

Next, we used Pearson correlations to assess the overall relationship between individual scores on the MWQ and trait-level variability on the ATQ, and then applied moderated regression analyses to probe into more specific links. We hypothesized that trait-level differences would play an important role in moderating the link between trait-levels of mind wandering and academic achievement, particularly for the Cognitive-Attentional trait of Effortful control. Across both analyses, given that the relationship between mind wandering, temperament traits, and academic achievement may be different for younger and older participants and across genders, we also examined whether our results varied by age and gender of participants. An *a priori* α level of 0.05 was used for all tests.

#### Correlation Analyses

**FIGURE 2:**
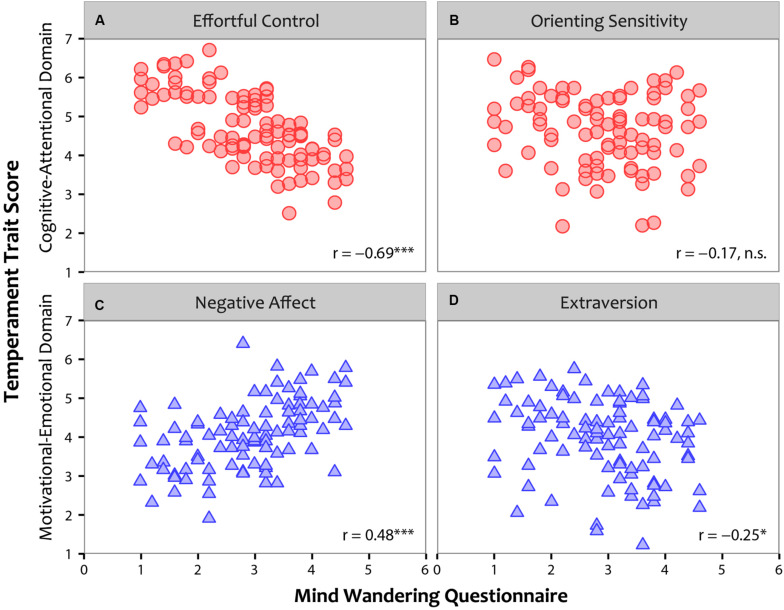
The relationship between individual participants’ scores on the Mind Wandering Questionnaire (MWQ; x-axis) and their temperament traits as assessed by the Adult Temperament Questionnaire (ATQ; y-axis). Cognitive-Attentional axes are depicted as red circles and Motivational-Emotional axes as blue triangles. Significant relationships are indicated as follows: *p < 0.05; **p < 0.01; ***p < 0.001.

Figure 2 illustrates the relationship between individual scores on the MWQ and the ATQ. Along the Cognitive-Attentional domain (depicted as red circles), reliable correlations emerged between mind wandering and Effortful control, *Pearson r*(95) = –0.69, *p* < 0.001, with greater trait-level Effortful control associated with lower levels of mind wandering (Figure 2A). No similar relationship emerged between mind wandering and Orienting sensitivity, *r*(95) = –0.17, *p* = 0.101 (Figure 2B). Along the Motivational-Emotional domain (depicted as blue triangles), both Negative affect, *r*(95) = 0.48, *p* < 0.001, and Extraversion, *r*(95) = –0.25, *p* = 0.015, related reliably to mind wandering, with higher Negative affect (Figure 2C) and lower Extraversion (Figure 2D) associated with higher mind wandering.

To examine whether these relationships varied by age and gender of participants, partial correlations were run between individual scores on the MWQ and the ATQ while controlling for age and gender. No changes were observed in the pattern of results, such that reliable correlations emerged between mind wandering and Effortful control, *Pearson r*(93) = –0.67, *p* < 0.001, Negative affect, *r*(93) = 0.48, *p* < 0.001, and Extraversion, *r*(93) = –0.25, *p* = 0.013, with no relationship between mind wandering and Orienting sensitivity, *r*(93) = –0.14, *p* = 0.19. Thus, neither age nor gender had a significant influence in controlling the relationship between mind wandering and temperament traits. As such, the data show robust links between individual propensity for mind wandering and both cognitive-attentional and motivational-emotional^1^ temperament traits.

#### Regression Analyses

**FIGURE 3:**
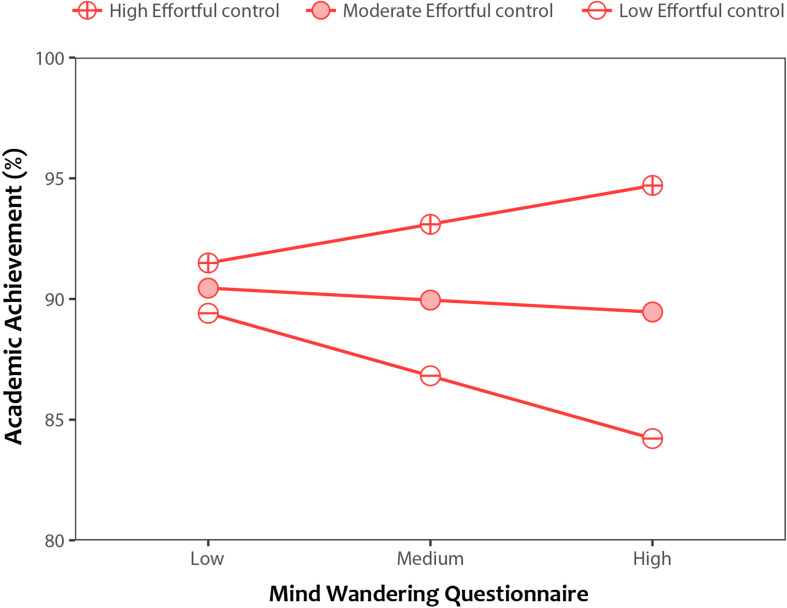
An interaction plot depicting the relationship between trait scores on the Mind Wandering Questionnaire and academic achievement, as moderated by the temperament trait of Effortful control. Simple slopes illustrate the moderating effect of high (+1SD above the mean), moderate (mean), and low (–1SD below the mean) Effortful control.

Here, we used hierarchical multiple regression analyses to examine whether individual temperament traits moderated the effect of mind wandering on the real-life outcome of academic achievement. When examining Effortful control as a moderating variable, scores on mind wandering and Effortful control were entered as predictors of academic achievement in the first step. These variables accounted for 10% of the variance in academic achievement, *R*^2^ = 0.10, *F*(2, 94) = 5.01, *p* = 0.009. To avoid potential issues with multicollinearity within the interaction term (Aiken and West, 1991), the variables were mean centered and an interaction term between mind wandering and Effortful control was created. Next, the interaction term between mind wandering and Effortful control was added to the regression model, and it accounted for an additional 4% of the variance in academic achievement, Δ*R*^2^ = 0.04, Δ*F*(1, 93) = 4.01, *p* = 0.048, *b* = 2.43, *t*(93) = 2.00, *p* = 0.048. An interaction plot, depicted in Figure 3, shows an enhancing effect of Effortful control on academic grades. For individuals with high Effortful control, higher tendency for mind wandering was associated with higher academic achievement, whereas for those with low Effortful control, higher tendency for mind wandering was associated with lower academic achievement.

For the other temperament traits, although mind wandering and Orienting sensitivity accounted for 7% of variance in academic achievement in the first step, *R*^2^ = 0.07, *F*(2, 94) = 3.28, *p* = 0.042, once the interaction term was added to the model, no moderation was found, Δ*R*^2^ = 0.001, Δ*F*(1, 93) = 0.14, *p* = 0.71, *b* = 0.43, *t*(93) = 0.38, *p* = 0.71. In addition, neither of the two temperament traits in the Motivation-Emotional domain were significant predictors of academic achievement in the first step [Negative affect, *R*^2^ = 0.06, *F*(2, 94) = 2.73, *p* = 0.07; Extraversion, *R*^2^ = 0.05, *F*(2, 94) = 2.63, *p* = 0.08], therefore moderation analyses were not pursued.

To examine whether our results varied by age and gender of participants, we conducted a follow-up hierarchical regression analysis to examine whether Effortful control moderated the effect of mind wandering on academic achievement when age and gender are added as covariates in the model. The model accounted for 14% of the variance in academic achievement, *R*^2^ = 0.14, *F*(5, 91) = 2.99, *p* = 0.015, with the interaction term between mind wandering and Effortful control accounting for 4% of this variance, Δ*R*^2^ = 0.04, Δ*F*(1, 91) = 4.23, *p* = 0.043, *b* = 2.52, *t*(91) = 2.06, *p* = 0.043; however, neither the age nor gender covariate was a significant explanatory variable [age, *b* = 0.08, *t*(91) = 0.81, *p* = 0.42; gender, *b* = –0.77, *t*(91) = 0.38, *p* = 0.70].

### Discussion

Experiment 1 examined the links between mind wandering, temperament traits, and the real-life outcome of academic achievement. The data indicated a relationship between individual variability on both cognitive-attentional and motivational-emotional temperament traits and individual propensity for mind wandering. Specifically, lower Effortful control, higher Negative affect, and lower Extraversion were linked to higher mind wandering. This dovetails with past research, which has linked mind wandering to external distractibility (Forster and Lavie, 2014, 2016), negative behavioral outcomes (Smallwood et al., 2008; Killingsworth and Gilbert, 2010; Smallwood and O’Connor, 2011), and other personality dimensions (i.e., extraversion, openness to experience) (Jackson et al., 2013; Smeekens and Kane, 2016; Kane et al., 2017). Further, and consistent with our hypotheses, the temperament trait of Effortful control emerged as the only reliable moderator of the relationship between mind wandering and the real-life outcome of academic achievement, with levels of effortful control regulating the effect of mind wandering on academic achievement. Additionally, all effects were stable across age and gender of participants.

Together these results conceptually replicate and extend previous work that has linked mind wandering with real-world educational outcomes like classroom fidgeting (Carriere et al., 2013). Importantly, however, over and above these past associations, the present data show that biologically primed temperament traits may play a regulatory role in modulating the real-life functional outcomes that are associated with mind wandering. As such, our findings are consistent with the notion that cognitive-attentional temperament traits are useful in predicting the real-life markers of mind wandering. We elaborate on these relationships further in the General Discussion.

Given that the link between mind wandering and effortful control was established using a self-report measure of mind wandering, in Experiment 2, we sought to replicate this data pattern by assessing if a similar relationship between mind wandering and effortful control is also found when mind wandering is experimentally elicited and objectively measured using a computerized task.

## Experiment 2

Although mind wandering is often assessed using self-report measures (Barron et al., 2011; Baird et al., 2012; Franklin et al., 2013), computerized tasks, such as the sustained attention to response task (Robertson et al., 1997) and the metronome response task (Seli et al., 2013b), can be used to provide an objective assessment of mind wandering.

Here, we used the visual version of the metronome response task (Laflamme et al., 2018). This procedure is an analog of the auditory metronome response task (Seli et al., 2013b) in which participants are asked to press a key synchronously to the onset of a repetitive tone. In the visual version, participants are presented with a visual target and asked to press a key synchronously with the appearance of that target. Variance in response times is taken as a measure of mind wandering, such that participants showing larger variance in responses are seen as having greater proportions of mind wandering during the task (Seli et al., 2013a, 2014, 2015b,c).

### Materials and Methods

#### Participants

One hundred and three participants were recruited via student volunteer participant pool. The data from ninety participants (10M, 80F; M_age_ = 21yrs, SD_age_ = 3yrs), who completed the study in full, were analyzed^3,4^. Written informed consent was obtained from each participant in accordance with the Declaration of Helsinki. All procedures were approved by the University Research Ethics Board. Each participant was remunerated with course credits.

#### Apparatus, Stimuli, and Design

Participants first completed the visual metronome response task (Laflamme et al., 2018). Then, they reported their demographic information completed the Mind Wandering Questionnaire (Mrazek et al., 2013), and the short-form Adult Temperament Questionnaire (Evans and Rothbart, 2007). The task took approximately 45 min. The study was administered online.

##### Visual metronome response task (vMRT)

In the vMRT, participants were presented with a central gray square (RGB [162, 162, 162], 2 × 2cm) shown against a white screen. They were instructed to press the space bar key at the same time that the gray square appeared, with the task sequence alternating between the presentation of a white screen (RGB [255, 255, 255]) for 1,150 ms and the presentation of the target gray square for 150 ms. Nine hundred trials were run. The task was coded in Javascript using jsPsych, a library developed to run behavioral experiments in a web browser (de Leeuw, 2015), which has been documented to have similar temporal resolution as in-lab studies (de Leeuw and Motz, 2016; Pinet et al., 2017).

### Results

Rhythmic response times (RRTs) were calculated by subtracting the onset time of the target gray square from the time that participants responded via keypress (Seli et al., 2013a, b, 2014, 2015b,c; Laflamme et al., 2018). This difference can either be negative if the participant responded prior to target presentation or positive if the participant responded after target presentation. Mind wandering was indexed as variance in mean RRT (ms^2^). This measure was computed by first calculating the RRT for each trial and then using a moving window technique to calculate the average variance of the current and preceding four trials across all trials throughout the task. As per previous studies (Seli et al., 2013b; Laflamme et al., 2018), each variance measure was adjusted using a natural-logarithm^3^ transformation to correct for the positive^4^ skew in the data.

Similar to Experiment 1, we used Pearson correlations to examine the relationship between individual participant’s scores on the vMRT and their scores on the ATQ dimensions, and then examined whether these relationships varied as a function of age and gender of participants.

#### Correlation Analyses

**FIGURE 4:**
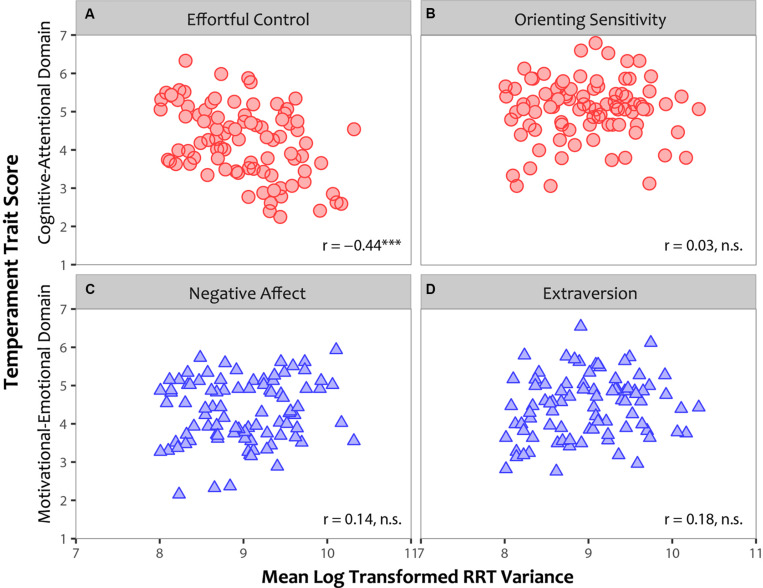
The relationship between individual participants’ mean log transformed variance in rhythmic response time (RRT) for the visual metronome response task (vMRT; x-axis) and their temperament traits as assessed by the Adult Temperament Questionnaire (ATQ; y-axis). Cognitive-Attentional axes are depicted as red circles and Motivational-Emotional as blue triangles. Significant relationships are indicated as follows: *p < 0.05; **p < 0.01; ***p < 0.001.

Figure 4 shows the correlations between individual participants’ mean RRT variance on the vMRT and the temperament traits of the ATQ. These data once again support the link between Effortful control and mind wandering. As in Experiment 1, there was a reliable negative correlation between mind wandering and Effortful control, *Pearson r*(88) = –0.44, *p* < 0.001 (Figure 4A), such that individuals with lower Effortful control mind wandered more on the vMRT. No significant relationships were found between mind wandering and Orienting sensitivity, *r*(88) = 0.03, *p* = 0.76 (Figure 4B), Negative affect, *r*(88) = 0.14, *p* = 0.21 (Figure 4C), or Extraversion, *r*(88) = 0.18, *p* = 0.08 (Figure 4D).

To examine whether these relationships varied by age and gender of participants, we controlled for age and gender by performing partial correlations between individual participants’ scores on the vMRT and the ATQ. As before, reliable correlations emerged between mind wandering and Effortful control, *Pearson r*(86) = –0.47, *p* < 0.001, with no relationship between mind wandering and Orienting sensitivity, *r*(86) = 0.03, *p* = 0.79, Negative affect, *r*(86) = 0.15, *p* = 0.18, or Extraversion, *r*(86) = 0.16, *p* = 0.14. Thus, as in Experiment 1, there was no change in the pattern of results observed when we controlled for age and gender of participants.

Thus, when we assessed mind wandering using an experimental measure, we once again found a reliable negative correlation between the trait of Effortful control and mind wandering, such that individuals with low Effortful control demonstrated greater variability in their responses in the visual metronome response task, indicative of greater mind wandering.

### Discussion

The results from Experiment 2 replicated Experiment 1 in that a reliable link was found between an objective measure of mind wandering obtained using the visual metronome response task and the temperament trait of Effortful control. This effect did not vary with participants’ age and gender. As such, they show that both self-report and objective measures of mind wandering relate to temperament traits that are reflective of cognitive and attentional domains.

However, it is important to note that the data in Experiment 2 also departed from the data in Experiment 1 in two ways. One, the links between mind wandering as assessed by the visual metronome response task and traits along the motivational-emotional axis were not reliable, such that both Negative affect and Extraversion did not vary with performance on the vMRT. Two, performance on this task also did not relate to the Mind Wandering Questionnaire, as there were no reliable correlations between mean RRT variability and responses on the MWQ, *Pearson r*(88) = 0.16, *p* = 0.13. As such, these differences suggest that different measures of mind wandering may relate to temperament traits differentially. It is possible that self-report measures of mind wandering may capture sensitivities within individuals that reflect stable behavioral processes across multiple domains, whereas laboratory measures of mind wandering may capture task-specific sensitivities that are best reflected across attentional domains.

In order to support these possible distinctions, we used the Fisher *r*-to-*z* transformation to compare the correlations between Effortful control and mind wandering from the two experiments. This analysis yielded a reliable difference such that correlations between Effortful control and mind wandering were significantly higher when mind wandering was measured using self-report versus an experimental paradigm, *z* = –2.50, *p* = 0.012. Future studies are needed to assess how differences in the ways in which mind wandering are measured, i.e., using subjective or objective measures, may be differentially related to stable temperament traits along the cognitive and/or affective dimensions.

## General Discussion

In this study, we investigated the influence of stable temperament traits on mind wandering and real-life performance. To do so, in Experiment 1, we assessed self-reported mind wandering, temperament traits, and academic grades in a large representative community sample. We found that individual differences in trait-levels of Effortful control, Negative affect, and Extraversion were independently related to self-reported mind wandering, with lower Effortful control, higher Negative affect, and lower Extraversion associated with higher reported rates of mind wandering. The temperament trait of Effortful control significantly moderated the relationship between mind wandering and academic success. That is, for individuals with high Effortful control, a higher tendency to mind wander was associated with higher academic achievement, and conversely for those with low effortful control, a higher tendency to mind wander was associated with lower academic achievement. In Experiment 2, we further demonstrated that the link between the trait of Effortful control and mind wandering was not dependent on participants’ self-reported measure of mind wandering, as this link was also reliable when mind wandering was measured using an objective experimental task. All effects were also stable even when controlling for covariates across age and gender of participants. Taken together, these results show that temperament traits along the cognitive-attentional axes can be used to predict both self-reported and objective behavioral markers of mind wandering, and as such may be an important regulator of the impact of mind wandering on real-world outcomes.

Extending previous work that has linked mind wandering with individual variability in cognitive abilities, the results from Experiment 1 show a nuanced relationship between innate individual differences, mind wandering, and functional behavioral outcomes. Researchers have long intuited that individuals with higher working memory capacity (Kane et al., 2007; McVay and Kane, 2012), higher ability to focus attention (Unsworth et al., 2012), and lower external distractibility (Forster and Lavie, 2014, 2016) are more effective at controlling their attention in order to successfully perform a task. More recent work examining links between personality factors and mind wandering identified pervasive personality influences as long-standing factors that affect an individual’s propensity to mind wander (Smeekens and Kane, 2016; Kane et al., 2017). Although it is perhaps intuitive that individuals who are more focused mind wander less, our paper shows that this link in part reflects the operation of innate regulatory influences that play a role in determining how mind wandering manifests within individuals and how it in turn impacts real-world functional outcomes. As such, our data extend previous work by demonstrating that individual variability in underlying temperament traits exerts a potential broad influence on behavior that may account for past results that have previously linked individual differences in cognitive processes and task control (Unsworth et al., 2012; Forster and Lavie, 2014, 2016; Smeekens and Kane, 2016; Kane et al., 2017), particularly since temperament traits are thought to mediate both the breadth and the stability of behavioral outcomes independent of external influences (Gagne, 2001; Rothbart, 2007).

Our data also revealed that the temperament trait of Effortful control was the only consistent influencing factor when examining mind wandering across both self-report scores and objective behavioral measures. Furthermore, Effortful control was the only trait to moderate the relationship between mind wandering and real-world functional outcomes of academic achievement, situating this temperament trait as a cognitive factor that may be able to significantly influence the functional consequences of mind wandering. Effortful control is purported to reflect intrinsic biologically-based differences in focusing attention on a task as a measure of attentional control (Rothbart et al., 2003; Rothbart, 2007). In addition, it also captures aspects of inhibitory and activation control, which respectively reflect an individual’s capacity to suppress unnecessary behavior and their ability to perform an action even with a strong tendency to avoid it. Given the broad range of this trait in capturing persistent cognitive and attentional styles, Effortful control appears to represent a larger overarching regulatory factor that may modulate the activity of mind wandering and attentional states. Thus, it may be possible that Effortful control moderates the relationship between mind wandering and real-world outcomes by controlling how each individual’s mind wanders for a given task at hand. For example, previous work has demonstrated that mind wandering can be purposefully allocated depending on task demands (Forster and Lavie, 2014; Seli et al., 2015a, 2017; Golchert et al., 2017). Thus, it may be possible for individuals with high Effortful control to flexibly shift their mind wandering from being dispersed in a stochastic and random manner to a strategic and deliberate manner during tasks that demand more cognitive resources, such as problem solving or engaging in social interactions. This reasoning dovetails with our finding showing that even if individuals high in Effortful control mind wander more, they are nevertheless likely to show better functional outcomes (i.e., higher academic performance) than individuals who are low in Effortful control, suggesting that this factor may be able to direct how mind wandering is distributed within tasks.

Further, these findings also suggest that individuals high in Effortful control may be distinct from those with low Effortful control across a number of real-world performance markers. Given results linking increased mind wandering with lower academic performance (Lindquist and McLean, 2011; Risko et al., 2012; Unsworth et al., 2012; Wammes et al., 2016), this knowledge can be beneficial for tailoring training and educational practices during early infancy and childhood when the expression of temperament traits is more malleable (Rothbart, 2007; Ganiban et al., 2008). Recent data support these efforts by showing that the strength of connections between the brain structures implicated in mind wandering and those associated with attention is related to differences in the real-life outcome of reading competence (Koyama et al., 2011). Thus, future work is needed to investigate the generality of the trait of Effortful control in capturing the diversity of attentional and mind wandering behaviors across a broader range of real-world outcomes.

Additionally, mind wandering and attentional behaviors may also relate differently *within individuals*. Specifically, within-participant variability in the regulatory control between mind wandering and attentional states, or the degree of fluctuations between off-task and on-task processes, may also inform functional outcomes. Research has long determined that attentional abilities fluctuate over the course of a day (Busch and VanRullen, 2010), over the span of an hour (Smallwood et al., 2008; Reichle et al., 2010; Terhune et al., 2017), and even over the span of minutes (e.g., 5 min; Cheyne et al., 2009). Although this evidence is suggestive of a recurrent pattern of attentive and mind wandering behaviors within individuals, the characterization of these variations and their effects on performance remain unknown. Understanding these variations and their sources will lead to a more comprehensive account of why mind wandering can exert both negative and positive effects on performance, behavior, and cognitive functioning (e.g., detriments in memory retention vs. benefits to creativity and future planning) (Smallwood and Andrews-Hanna, 2013).

Finally, although this work yielded robust results and found a similar relationship between mind wandering and Effortful control using both self-report and objective lab-based measures of mind wandering, Effortful control accounted for a relatively small amount of variance in the regression model in Experiment 1. This is likely due to the fact that the current work focused on the links between mind wandering, temperament traits, and the real-world outcome of academic achievement. As such, it may be possible to capture more variability in the data by including additional outcomes within the model that have been associated with mind wandering, such as reading comprehension, working memory, or general mood (Mooneyham and Schooler, 2013). Alternatively, future work could also examine how the relationship between mind wandering and temperament traits may vary across different contexts, such as laboratory tasks versus daily activities (Kane et al., 2017).

In sum, in this study, we demonstrated links between the stable biologically primed temperament trait of Effortful control and mind wandering, which we assessed using both self-reported and objective experimental measures. We also demonstrated that individual trait-level ability to control attention modulates the relationship between mind wandering and the real-life outcome of academic success. This finding highlights temperament as one of the stable underlying factors that may drive, modulate, and sustain attentional behaviors in the long-term.

## Data Availability Statement

The data from our article are publicly available via open science repository, which can be accessed here: https://osf.io/53zd9/.

## Ethics Statement

The studies involving human participants were reviewed and approved by McGill University Research Ethics Board. All participants provided their written informed consent to participate in this study.

## Author Contributions

EP and JR developed the initial study concept. EP and LG implemented the study and performed data collection. All authors were involved in design, analyses, interpretations, manuscript preparation, and final approval of the manuscript.

## Conflict of Interest

The authors declare that the research was conducted in the absence of any commercial or financial relationships that could be construed as a potential conflict of interest.

## Footnotes

1 An *a priori* power analysis was performed for sample size estimation, and determined that with an α = 0.05 and power = 0.8, the projected sample size needed to detect a small effect of 0.3 (Evans, 1996) was 84 participants. Our proposed sample size also included additional participants to account for expected attrition and quality control (3–34%, as per Peer et al., 2014).

2 First, all data were examined for completeness and quality. Participants who did not complete all required components (i.e., the MWQ, ATQ, and Percent Grade) were excluded from the analyses (*N* = 28). The responses from the remaining participants were examined for quality by assessing systematic errors in responding to reverse-scored questions (e.g., *“I never have difficulty focusing my attention on boring and mundane tasks”*). No participants were excluded based on this criterion. Finally, data were also examined for outliers, and three participants were excluded based on their reported academic grades falling 3 standard deviations below the group mean.

3 *A priori* power to detect a small effect was equivalent to power estimates in Experiment 1.

4 As in Experiment 1, data were examined for completeness, quality, and any systematic errors in responding to reverse-scored questions. Two participants were excluded based on these criteria. As per Seli et al. (2013b) recommendations, any participants with rates of omissions (e.g., failure to respond on any given trial) greater than 10% were excluded from subsequent analyses. Eleven participants were excluded based on this criterion.
